# Femoral head cartilage reconstruction using autologous osteochondral mosaicplasty: A case report

**DOI:** 10.1097/MD.0000000000032913

**Published:** 2023-02-10

**Authors:** Hyeonjoon Lee, Hyoung Tae Kim, Suenghwan Jo

**Affiliations:** a Department of Orthopedic Surgery, Chosun University Hospital, Gwangju, South Korea; b Chosun University, School of Medicine, Gwangju, South Korea.

**Keywords:** autologous bone graft, cartilage injury, femoral head, hip dislocation, mosaicplasty, osteoarthritis, osteochondral autograft transfer

## Abstract

Cartilage injuries of the femoral head may occur following hip dislocation. As a rare injury, controversy persists regarding ideal treatment of damaged femoral head cartilage. Here we report the case of a patient who developed a large cartilage injury to the femoral head following anterior hip dislocation for which autologous osteochondral mosaicplasty with a graft harvested from the ipsilateral femoral head achieved a satisfactory outcome.

**Patient concerns:**

A 62-year-old man developed a right hip dislocation after a fall from a 5-m height and was referred to our institution.

**Diagnoses:**

The initial diagnosis was anterior hip dislocation. Upon hip joint reduction, a simple radiograph and computed tomography scan showed a large cartilage defect in the superolateral region of the femoral head. Multiple bony fragments were visible within the joint.

**Interventions:**

The hip joint was surgically dislocated. The large cartilage defect of the femoral head was treated with autologous mosaicplasty using an osteochondral autograft transfer system using multiple osteochondral plugs retrieved from a non-weight-bearing portion of the ipsilateral femoral head.

**Outcomes:**

Diagnostic hip arthroscopy performed at 8 months postoperative confirmed full incorporation of the osteochondral graft into the native femoral head. At the 2-year follow-up, the patient was pain-free, had a normal range of motion and displayed no evidence of osteoarthritis.

**Lessons:**

Isolated femoral head cartilage injuries may occur as a consequence of anterior hip dislocation. A femoral head with a large irregular cartilage defect can be treated with mosaicplasty using an osteochondral autograft from a non-weight-bearing portion of the ipsilateral femoral head.

## 1. Introduction

Fracture of the femoral head is a rare injury that may occur as a consequence of the femoral head impacting the acetabular wall or from shear force during dislocation.^[1,2]^ As the hip joint is intrinsically stable, such injuries usually occur only after high-energy trauma; the incidence is reportedly 5–15% following posterior hip dislocation.^[3,4]^ Due to their rare nature, controversy persists regarding their treatment. Here we report the case of a patient who developed a large cartilage injury to the femoral head following anterior hip dislocation. Autologous osteochondral mosaicplasty was performed using grafts harvested from the ipsilateral femoral head. Incorporation of the transferred autograft was confirmed through arthroscopy, and the result was satisfactory at 2 years postoperative.

## 2. Case presentation

A 62-year-old man was referred to our emergency department with right hip pain that developed after a 5-m fall from a tree. He was initially examined by a general practitioner in a local institution and transferred to our institution immediately following the diagnosis of hip dislocation. The patient had a distal radial fracture on the left wrist 13 months prior to the current injury that had been fixed with a titanium plate. The patient had no other relevant history and was an independent ambulator prior to the injury.

**Figure 1.**
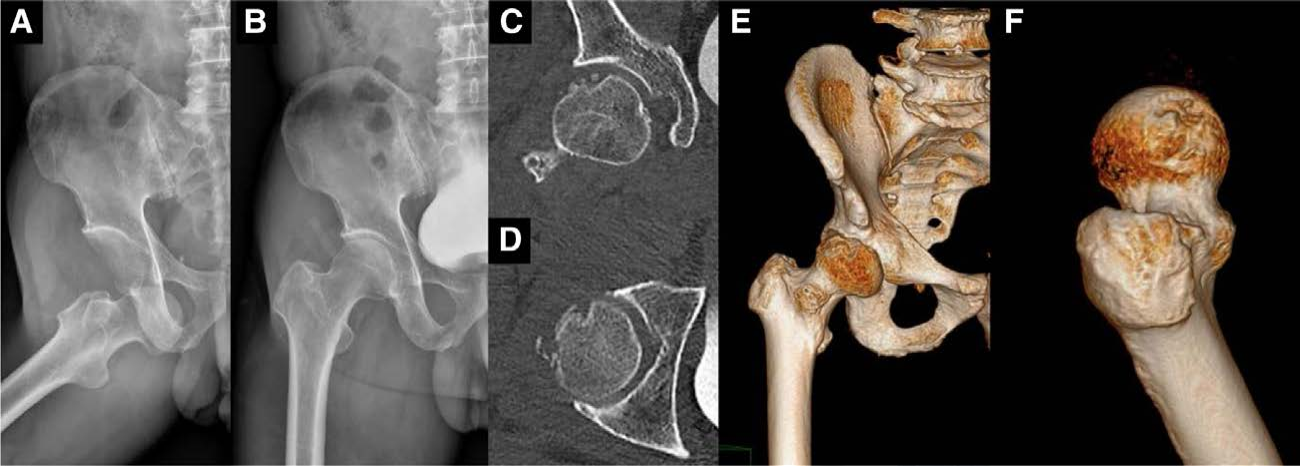
(A) Pelvic X-ray showing the hip outside the socket while in external rotation indicating an anterior dislocation. (B) The reduction was successful but a small bony defect is visible in the superior lateral femoral head. A femoral head–impacted fracture with a large cartilage defect is visible on coronal (C), axial (D), and 3-dimensionally reconstructed (E, F) computed tomography images.

At the initial presentation, the patient’s right leg appeared deformed with the hip externally rotated and extended. His mental status was alert, but he reported severe pain in the right hip with a visual analog scale score of 9. A large bruise was noticed on the right lateral thigh with multiple abrasions, and the motion of the right hip was limited. The motor and sensory functions of both lower legs were normal, and he reported no tenderness elsewhere. All results from routine laboratory tests were within the normal range. Simple radiographs of the pelvis revealed that the hip was in an anteriorly and medially dislocated position. No other evident fractures were noted at the time of the initial imaging examination (Fig. 1A). He was immediately sent to the operating room for reduction using the Allis maneuver under general anesthesia. Briefly, the patient lay supine on the surgical table, and the dislocated hip was placed in 90 degrees of flexion. While an assistant stabilized the pelvis against the bed, the hip was placed in traction in line with the femur. After the clunk sensation was noted in the hip, the leg was internally rotated to complete the reduction. With the patient still under anesthesia, the leg’s range of motion and stability were checked, and no limitation or instability was noted. Eight hours passed between the initial injury and the reduction.

Simple radiography of the pelvis following the reduction confirmed the femoral head in its anatomical position. However, a bony defect resembling an erosion was noted in the superolateral region (Fig. 1B). Computed tomography performed to further evaluate the status of the femoral head showed a large cartilage defect in the superolateral portion of the femoral head with an estimated defect size of 20 mm × 23 mm (Fig. 1C–F). Multiple small bony fragments were visible within the hip joint. A small bony deformation in the anterior acetabulum suggested that the femoral head impacted the anterior rim of the acetabulum and had caused the cartilage injury. The status of soft tissue, such as the ligamentum teres or anterior labrum, was not evaluated at this time. Multiple bony fragments within the joint were too small to fix, and a plan was made to excise them to prevent secondary osteoarthritis. Regarding the cartilage defect on the femoral head, after a thorough discussion with the patient, the decision was made to perform cartilage transplantation. This option was chosen based on the likelihood of secondary osteoarthritis with nonoperative treatment and the patient’s desire to preserve the native hip joint.

**Figure 2.**
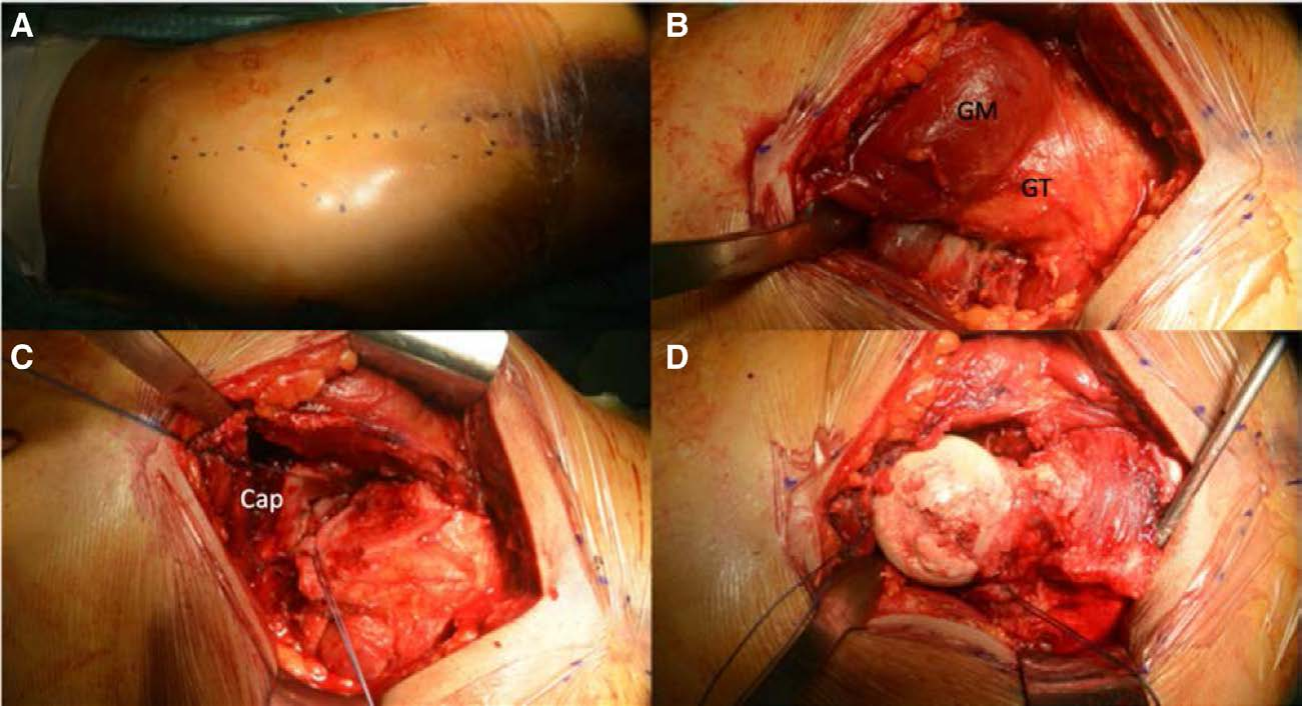
(A) A skin incision was made centered at the GT as described by Ganz et al.^[5]^ (B) The posterior portion of the GT was exposed for a trochanteric osteotomy. (C) The anterior and superior capsule had been disrupted by the dislocation. (D) The dislocated femoral head displayed a larger cartilage defect on the weight-bearing portion. Cap = capsule, GM = gluteus medius, GT = greater trochanter.

Surgical intervention was performed 3 days after the injury. The hip joint was approached using surgical dislocation as described by Ganz et al^[5]^ (Fig. 2). Briefly, a 10-cm incision was made longitudinally centered at the lateral trochanter, and the tensor fascia lata was split. With the hip in internal rotation, the posterior aspect of the greater trochanter was exposed and a trochanter osteotomy was performed using an oscillating saw. This process preserves the posterior short rotator complex, thereby leaving the posterior vascular structures undisrupted. The capsule had been partially disrupted by the previous dislocation, and the ligamentum teres was disrupted. The hip was externally rotated to dislocate the femoral head from the acetabulum.

**Figure 3.**
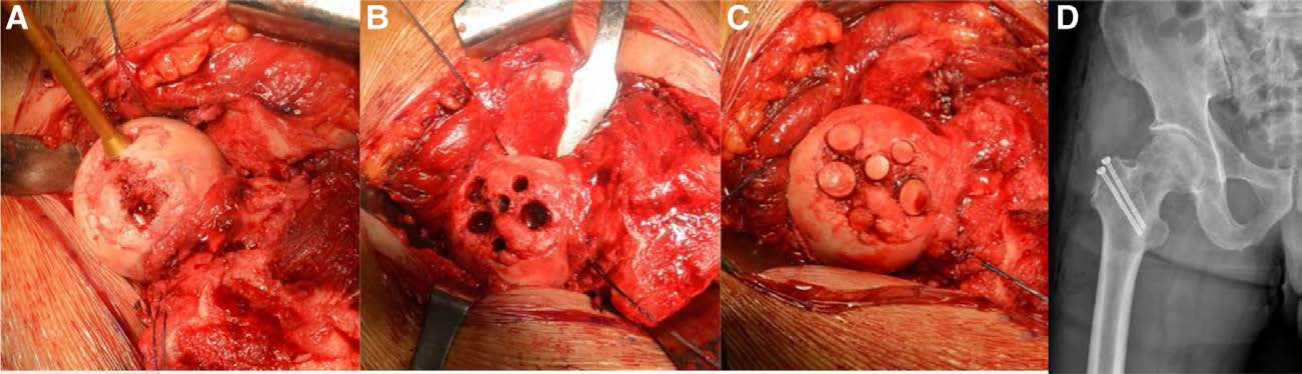
(A) Measuring the cartilage defect with the osteochondral autograft transfer system sizer. (B) Preparing to adopt the osteochondral graft transfer. (C) View after the mosaicplasty was performed. (D) Postoperative X-ray following the autologous mosaicplasty.

The weight-bearing portion of the exposed femoral head showed a 35 × 27 mm cartilage defect, larger than that estimated using computed tomography. Due to its large size, mosaicplasty was performed using an osteochondral autograft transfer system (Loaner Allograft instrument set; Arthrex, Naples, FL, USA). After marginal debridement of the defective cartilage, the appropriate osteochondral cartilage graft sizes were measured using an osteochondral autograft transfer system sizer. Because of the defect’s large and irregular shape, at least 6 osteochondral plugs of varying diameters (3 × 10 mm, 2 × 6 mm, and 1 × 8 mm) were required to cover the cartilage-defective region. A decision was made to use no more than 6 osteochondral plugs, as the distal portion of the plug may interrupt each other during the impaction process. The hip was further externally rotated to expose the inferior non-weight-bearing portion of the femoral head, and 10-mm-long osteochondral plugs of predetermined size were harvested. The height of the harvested autografts was adjusted to maintain the congruent shape of the femoral head, and the donor grafts were press-fitted into the prepared recipient sites. Following the mosaicplasty, the hip was reduced, the capsule repaired, and the osteotomized greater trochanter was fixed with two 4.5-mm cannulated screws (Fig. 3).

Following surgery, the patient was placed in a hip brace to restrict external rotation for 4 weeks. While the use of a wheelchair was allowed immediately after surgery, partial weight-bearing with crutches was allowed at 4 weeks and full weight-bearing without a crutch was allowed at 12 weeks postoperative. The main complaint of the patient following surgery was pain in the greater trochanter region where the osteotomy was performed. This lasted for 6 months but decreased over time. The inguinal pain was subtle and completely resolved within 3 months postoperative. The patient was able to walk without a crutch at 12 weeks postoperative, but limping persisted until the 20 weeks postoperative.

**Figure 4.**
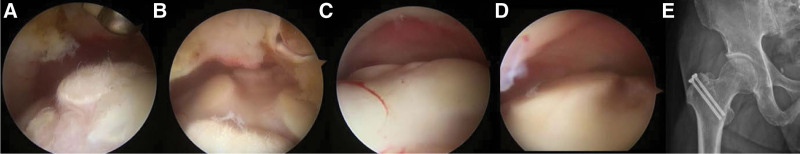
Arthroscopic findings of the grafted osteochondral autograft at varying angles (A–D). (E) Follow-up X-ray taken 2 years after the autologous mosaicplasty showing no signs of osteoarthritis.

The patient underwent diagnostic hip arthroscopy at 8 months after index hip surgery to confirm the status of the transferred osteochondral autograft. This was performed at the patient’s request, as he underwent planned implant removal surgery on the left wrist. Diagnostic hip arthroscopy was performed with the patient in the lateral decubitus position using a McCarthy hip retractor. Conventional anterior and anteromedial portals were used with a 70-degree scope inserted through the former. Arthroscopy revealed that the grafted bone was firmly incorporated into the native femoral head. While deficient cartilage was present peripheral to the grafted site, no evidence of osteoarthritis was observed in the grafted osteochondral cartilage, native femoral head cartilage, or acetabulum (Fig. 4).

The patient was followed up every 6 months until 2 years postoperative. His modified Harris Hip Score was 72 and his International Hip Outcome Tool score (iHOT-12) was 68 at 3 months; these values improved to 95 and 92, respectively, at the last follow-up. No evidence of osteoarthritis or osteonecrosis was found on radiography at the 2-year follow-up.

Ethical approval was waived as this is a report of a single case. Written informed consent was provided by the patient’s legal guardian concerning his case being submitted for publication.

## 3. Discussion

The decision to intervene in a fracture of the femoral head is largely based on fracture location and size and treatment often consists of simply removing the loose bodies and debriding the cartilage.^[6]^ Such treatment may yield a satisfactory outcome when the injury is located in the non-weight-bearing region, but it may result in secondary osteoarthritis if it occurs in the weight-bearing zone or involves concomitant loose body remnants.^[7]^ Therefore, when the fracture is accompanied by extensive cartilage damage, surgical restoration should be considered to prevent further joint destruction. However, although the treatment methodology for cartilage injuries of the knee and ankle joints is well established, such injury in the femoral head is rare and optimal treatment is controversial.^[8]^ The cartilage restoration strategies used in other joints include microfracture, osteochondral transfer using an allograft or autograft, and autogenous chondrocyte implantation. Of these options, autologous graft transplantation, first reported by Hangody et al,^[8]^ has been widely utilized in the knee, ankle, and shoulder joints with favorable outcomes. However, the application of this method to the hip joint is limited because of rarity and the complexity of approaching the joint. Nonetheless, when optimally performed, cartilage restoration can be expected through the formation of hyaline cartilage, which may provide a promising outcome.

In the current case, we chose to perform the osteochondral mosaicplasty because of the large size and irregular shape of the osteochondral defect. The challenge of mosaicplasty involves retrieving an osteochondral graft of adequate size and shape. While allografts can be considered, we decided to use autografts to prevent potential complications such as bone resorption or infection. Autologous osteochondral grafts are commonly harvested from the non-weight-bearing zone of the knee joint; however, donor-site morbidity is a reported complication.^[9]^ Considering that multiple osteochondral plugs were required for the current surgery, significant donor-site pain may have been caused. We were able to access the inferior portion of the femoral head using surgical dislocation and retrieve 6 osteochondral plugs that could cover the cartilage defect. Compared to harvesting osteochondral plugs from the knee joint, this method features a shorter surgical time and prevents donor-site morbidity. This approach also preserves the posterior vascular structures; therefore, potential complications from a damaged vascular supply, such as osteonecrosis, could be prevented.

A concern with mosaicplasty was that we were unable to cover the entire cartilage defect owing to its irregular shape. As we harvested 10-mm-long plugs to maximize the chance of osteointegration, completely filling the defective cartilage with the plugs may have caused the distal portions of the plugs to bump into each other, causing plug breakage. Therefore, we decided to use 6 plugs transferred 5 to 10 mm apart. The result was successful, as the plugs fully integrated into the native femoral head and secondary osteoarthritis prevented.

There are few reports on the application of osteochondral autografts to the femoral head. Won et al^[10]^ reported a case of femoral head fracture in which grafts were sized from the ipsilateral femoral head using osteochondral autograft transfer system. The defect in their case was relatively small, and the authors were able to restore the defect with a single plug of the osteochondral autograft. A case report by Kong^[11]^ reported an autograft harvested from the knee joint to restore the bone defect in the femoral head. Interestingly, the patient also developed osteochondral damage following anterior hip dislocation. The procedure was successful, but the author reported that it was technically demanding and required substantial operation time.

Based on this report, we suggest that mosaicplasty with an osteochondral plug harvested from a non-weight-bearing portion of the ipsilateral femoral head can be considered when a large cartilage defect of the femoral head is present. Surgery can be performed through surgical dislocation and has the advantage of a shorter operation time and preventing donor site pain. Even without restoring entire cartilage, this strategy provides a sufficient support to prevent osteoarthritis.

In summary, injury to the femoral head cartilage may occur as a consequence of anterior hip dislocation. Mosaicplasty using an osteochondral autograft from a non-weight-bearing portion of the ipsilateral femoral head can be considered when the femoral head develops a large irregular cartilage defect.

## Author contributions

**Conceptualization:** Suenghwan Jo.

**Funding acquisition:** Suenghwan Jo.

**Investigation:** Hyeonjoon Lee, Hyoung Tae Kim.

**Methodology:** Suenghwan Jo.

**Supervision:** Suenghwan Jo.

**Visualization:** Hyoung Tae Kim.

**Writing – original draft:** Hyeonjoon Lee, Hyoung Tae Kim.

**Writing – review & editing:** Suenghwan Jo.
